# Tracheal Stent Ingestion: Unveiling Complications and Innovations in Management

**DOI:** 10.14309/crj.0000000000001404

**Published:** 2024-07-19

**Authors:** Fnu Vikash, Osagiede Osayande, Maoyin Pang

**Affiliations:** 1 Department of Medicine, Jacobi Medical Center, Albert Einstein College of Medicine, Bronx, NY; 2 Division of Gastroenterology and Hepatology, Mayo Clinic, Jacksonville, FL

**Keywords:** tracheal stent, migration, endoscopy, foreign body, stomach

## Abstract

Airway stenting has become integral to the therapeutic endoscopic management of benign and malignant obstructive airway diseases. Despite the increased use of stents, the absence of clear guidelines for surveillance and maintenance poses the potential for unique stent-associated complications. Our case reports a rare incident of tracheal stent dislodgement, leading to its ingestion and unexpected discovery within the stomach. This case serves the purpose of shedding light on a rare yet potentially life-threatening complication and discussing types of stent and characteristics to enhance gastroenterologists' understanding of stent-related challenges and equips them to anticipate and strategize the appropriate course of action.

## INTRODUCTION

Intraluminal tracheobronchial stents are used to treat airway obstruction because of both malignant and benign obstructive diseases of the trachea or bronchi.^1,2^ In conjunction with the standard management for the underlying primary disease, stents relieve dyspnea and improve overall functional status.^3^ Stents come in various shapes and sizes and are made of various biocompatible materials, each with unique insertion techniques.^4,5^ However, the search for an ideal stent is still ongoing.^6^ Stents are foreign objects and are not devoid of complications.^5^ These include malposition, stent fractures, stent migration, airway perforation, excessive granulation-tissue formation, hemorrhage, bacterial colonization resulting in stent-associated infections, stent obstructions because of tumor, granulation tissue, and/or mucostasis.^5,7^

These complications may be life-threatening and hence may justify surveillance bronchoscopy at an interval of 4–6 weeks after placement.^7^ Studies report stent migration rates between 20% and 50%, whereas few studies report a much lower rate (<5%).^5,8,9^ A mismatch between the size of the airway and the stent diameter is believed to be one of the factors resulting in stent migration.^10^ In this article, we present a unique case wherein the displacement of a stent leads to its inadvertent ingestion during a coughing episode. This case represents only the second reported instance worldwide of swallowing a tracheal stent during a coughing episode.

## CASE REPORT

A 48-year-old man with a medical history notable for hypertension, non–insulin-dependent diabetes mellitus, and morbid obesity (body mass index = 47 kg/m^2^) was diagnosed with coronavirus disease 2019 pneumonia. The patient had prolonged hospitalization for 6 months, during which the patient required mechanical ventilation and venovenous extracorporeal membrane oxygenation.

The hospital course was complicated by severe tracheomalacia and tracheal stenosis, requiring tracheal resection and reconstruction. After the failure of the initial reconstruction, a tracheal stent was placed with venovenous extracorporeal membrane oxygenation support. Follow-up bronchoscopy revealed stent fractures, necessitating temporization with balloon dilation and the placement of a Y-shaped silicone stent to cover the AERO stent (Merit Medical Systems, Inc., South Jordan, UT) fracture.

**Figure 1.**
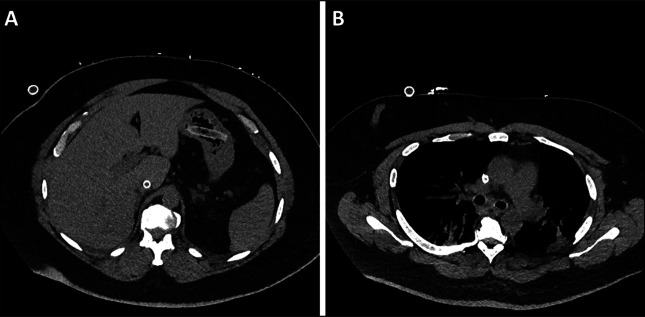
Abdominal CT without contrast shows the presence of a stent in the stomach (A); chest CT without contrast shows an absence of a stent (B). CT, contrast tomography.

**Figure 2.**
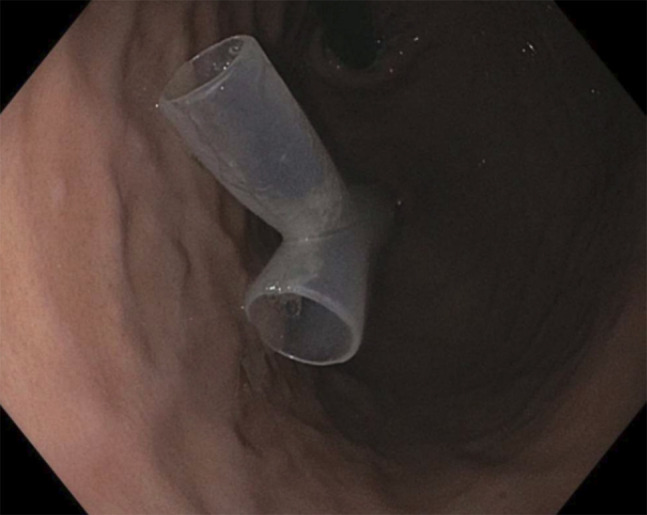
Y-shaped silicone stent in the gastric fundus.

On admission to the hospital for stent exchange, rigid bronchoscopy demonstrated the absence of a tracheal stent. The patient reported experiencing a severe episode of coughing and choking before the bronchoscopy. However, he denies any difficulty in swallowing; there were concerns about the possibility of coughing up and swallowing the stent. An abdominal and pelvic computed tomography scan without contrast revealed the presumed ingested tracheal stent within the gastric fundus without evidence of perforation, as shown in Figure 1. In response, emergent upper gastrointestinal endoscopy was performed, with retrieval of the intact Y-shaped silicone stent from the gastric fundus using a Roth Net device (US Endoscopy [a subsidiary of Steris Corporation], Mentor, OH) (Figure 2). The patient underwent a successful replacement of a Y-tracheal stent the following day and was subsequently discharged from the hospital after recovery. There was no evidence of tracheoesophageal fistula based on computed tomography scan, bronchoscopy, and endoscopy.

## DISCUSSION

This case report outlines an uncommon and captivating occurrence involving the dislodgment of a tracheal stent during a coughing spell resulting in ingestion. Stents are broadly classified into 2 groups, including plastic (eg, silicone) and metallic stents (eg, stainless steel). Several complications of tracheal stents have been described (Table 1). The primary complications arising from human errors are typically associated with stent misplacement, incorrect sizing, and overly extended positioning.^11^ The most frequent complications related to stents include migration, fracture, patient intolerance, and the formation of granulation tissue around the stent.^12^ In our case, attempts to manage tracheal stenosis through stenting proved unsuccessful. The initial stent experienced a fracture, and during the second attempt, it dislodged and was subsequently swallowed into the stomach.

**Table 1.** Types of stents, characteristics, complications, and notable features/innovative features

Stent type	Characteristics	Risk factors	Complications	Notable features/innovations
Plastic	Silicone or other polymers	Loose placement Stenting a short segment with smooth mucosa	Migration Fracture Patient intolerance Granulation tissue formation around the stent	Modification to a straight tube for easier insertion. Ability to be fixed to the airway
Metal	Stainless steel	Inadequate sizing Stenting a short segment with smooth mucosa	Migration Fracture Patient intolerance Granulation tissue formation around the stent	Rigidity and lack of flexibility
AERO	Newer generation with built-in features (eg, anti-migration fins)	Not specified	Fracture	Anti-migration fins
Ultra-flex	Metal stent (eg, Nitinol)	Not specified	Migration Fracture Patient intolerance Granulation tissue formation around the stent	Flexibility and ease of retrieval
Biodegradable	Degradable polymers (eg, polydioxanone)	Not approved by Food and Drug Administration in North America	Not specified	Degrades without extraction. Ongoing research, mainly focused on Europe

The tendency for stents to migrate differs between different stent types.^7,13,14^ When silicone T-tube stents were modified to a straight tube, the design allowed easier endoscopic insertion, less incidence of secretion plugging, and the added benefit of being more esthetically acceptable to patients since a stoma was no longer needed.^15^ Silicone stents can be fixed to the airway to reduce the possibility of migration. However, this anchoring option is not feasible for self-expandable metal stents (SEMS) because of their rigid nature and lack of flexibility.^16^ Other risk factors for stent migration include using a loose stent or stenting a short segment (≤2.5 cm) with smooth mucosa.^17,18^ Cough is the most common symptom of stent migration.

The ideal stent should (i) be easy to install, (ii) easily retrievable by bronchoscopy, (iii) difficult to dislodge during maneuvers that increase airway pressures, (iv) allow unimpeded mucus clearance, and (v) incite little or no mucosal irritation.^15^ As of now, the ideal stent has yet to be developed that has all of the desirable qualities. Perhaps, this ultimate combination will never be found, but efforts are ongoing. The newer generation AERO stents have built-in features such as antimigration fins and larger diameters toward the proximal and distal ends.^12,19^ Our patient initially had an AERO stent placed, which unfortunately got fractured, and the patient had to get a silicone stent as a temporizing measure. The biodegradable stents represent an innovation in the future of stent technology and practices. It is made of degradable polymers such as polydioxanone, and these stents degrade without requiring any extraction. The Food and Drug Administration has not approved the use of biodegradable stents in North America. In addition, no product patents have been filed with the Food and Drug Administration by any stent manufacturers in North America for its experimental use. Thus, biodegradable stents are a novel idea, with much of its ongoing research focused in Europe.^20^ Migration of SEMS remains a problem. When dislodged, SEMS can be ingested or cause asphyxiation if remains trapped in the hypopharynx.

This case highlights the importance of a multidisciplinary approach to address stent migration, requiring collaboration between pulmonologists and gastroenterologists. By illuminating this rare but life-threatening complication, it enhances gastroenterologists' understanding of tracheal stent-related challenges, enabling them to anticipate and strategize the appropriate course of action.

## DISCLOSURES

Author contributions: F. Vikash: chart review and writing a manuscript draft and revisions. O. Osayande: chart review and manuscript revisions. M. Pang: manuscript critical review and is the article guarantor.

Financial disclosure: None to report.

Informed consent was obtained for this case report.
